# Self-administration of aspirin for acute chest pain—Does it prevent premature cardiovascular mortality?

**DOI:** 10.1007/s00508-024-02471-w

**Published:** 2024-12-17

**Authors:** Gudrun Lamm

**Affiliations:** Department of Internal Medicine 3, Karl Landsteiner University of Health Sciences, Dunant Platz 1, 3100 St. Poelten, Austria

**Keywords:** Antiplatelet therapy, Myocardial infarction, Thrombolysis, Non-cardiac chest pain, Bleeding risk

## Abstract

Aspirin as a class I guideline recommended medical treatment for acute coronary syndrome has been established for decades [1]. As early intake of aspirin is crucial, self-administration of aspirin in acute chest pain might be beneficial when weighing up the potential harm including a slightly elevated bleeding risk in patients with chest pain of another origin than myocardial infarction against the benefit in patients with coronary ischemia.

## Introduction

Aspirin is an acetylated form of salicylic acid which is necessary for its antiplatelet effect. The analgesic effect of willow bark was already known at the time of Hippocrates [2]. Despite this drug being introduced for the treatment of inflammatory conditions in the 1890s, the antiplatelet effects were recognized in 1967 [3]. Aspirin was the first antiplatelet agent used in patients with acute myocardial infarction [4]. The major antiplatelet effect results from the irreversible (8–11 days) inhibition of the platelet cyclooxygenase‑1 by selective acetylation of a hydroxyl group on the prostaglandin H2 synthase leading to inhibition of thromboxane A2 dependent platelet function. Platelet-dependent hemostasis is resumed by megakaryocyte-dependent generation of new platelets.

Data suggest no clear benefit of aspirin in primary prevention [5–7]. In contrast, in secondary prevention in patients with established cardiovascular disease, the evidence for the use of aspirin is strong [8].

The second international study of infarct survival (ISIS-2) [9] demonstrated for the first time that the immediate administration of aspirin in patients with acute myocardial infarction results in a mortality reduction of more than 23%. Nevertheless, there are still some reasons to withhold this therapy in selected cases. With respect to bleeding complications, several studies have shown a favorable risk-benefit ratio in aspirin-treated patients when compared to aspirin non-users. Moreover, timing of administration is of utmost importance. The current European Society of Cardiology (ESC) guidelines on acute coronary syndrome [1] recommend starting aspirin as soon as possible. Thus, self-administration may guarantee the earliest possible time to start aspirin after onset of acute chest pain. This paper focuses on the balance of potential risks and life-saving benefits of aspirin self-administration.

## Strong evidence of benefit of aspirin in acute myocardial infarction – the earlier the better

The landmark trial showing benefit of aspirin in acute myocardial infarction was the ISIS-2 trial [9] published in 1988. More than 17,000 patients presenting with an acute myocardial infarction (median time from symptom onset to presentation 5 h) were randomized to streptokinase (1.5 mU for 1 h), aspirin (160 mg per day for 1 month), both or placebo. Not only streptokinase but also aspirin alone significantly reduced the 5‑week vascular mortality compared to placebo, and the combination of both further reduced vascular deaths (i.e. deaths due to cardiovascular causes). This survival benefit was preserved even after a follow-up of 15 months. Whereas streptokinase was associated with an excess of bleeding events including cerebral hemorrhage, aspirin did not increase the occurrence of excess bleeding requiring transfusions or cerebral hemorrhage but also significantly reduced non-fatal reinfarction and stroke. The excess rate of reinfarction within the first days in patients treated with streptokinase alone could be prevented by concomitant administration of aspirin. The American College of Cardiology and the American Heart Association recommended aspirin to be started immediately at a dose of 160 mg in all patients presenting with an acute myocardial infarction and to be continued indefinitely 2 years after publication of the ISIS‑2 trial [10].

Aspirin was introduced as a class I medical therapy in patients presenting with an acute myocardial infarction. Current European guidelines [1] recommend aspirin at a loading dose of 150–300 mg as soon as possible followed by a maintenance dose of 75–100 mg per day.

Nevertheless, prehospital administration of aspirin in patients with presumed acute coronary syndrome remains low. The US Emergency Medical System data demonstrate prehospital aspirin utilization in about 45% of patients with suspected acute coronary syndrome [11].

## Pharmacodynamics of aspirin and timing

Platelet inhibition occurs early and peaks within minutes after aspirin administration [12]. The dose of aspirin also plays a major role because higher doses lead to early peak aspirin levels compared to doses less than 100 mg.

Timing of aspirin administration is largely influenced by the mode of first contact. Data from the ERICO study [13] demonstrated that individuals presenting in primary care units received the first aspirin dose much earlier than those presenting in hospitals; however, only 75.6% of the patients with suspected acute coronary syndrome received the first aspirin dose in the primary care unit, the remaining patients were started on aspirin in the hospital.

In patients with acute myocardial infarction treated with thrombolysis the benefit of aspirin is also time dependent. When compared to late aspirin use, early use (median time from symptom onset 1.6h versus 3.5 h in early vs. late aspirin use, respectively) was associated with lower mortality at 7 days, 30 days and at 1 year [14].

A systematic review that included three studies (one randomized and two non-randomized controlled trials) suggested that early (median 1.6 h) or first-aid administration of aspirin in patients with presumed acute myocardial infarction or non-traumatic chest pain leads to improved survival at 7 days and at 1 year compared to late (median 3.5 h) or in-hospital administration of aspirin [15].

## Drug formulation and onset of action

Data measuring serum salicylate concentration at baseline and at 5–180 min after administration showed that chewable aspirin formulations are more rapidly absorbed compared to solid aspirin tablets (that is either swallowed as a whole or first chewed and then swallowed) [16].

Swallowing of oral aspirin solution results in even higher peak concentrations when compared to a chewed solid aspirin tablet. The plasma concentration of acetylated acid was significantly higher 3min after swallowing 300 mg aspirin in an oral solution compared to the same dose administered as a chewed solid tablet [17].

Intravenous application of aspirin in patients with acute coronary syndrome results in lower residual platelet aggregability at 5 min and 20 min when compared to oral aspirin; however, no difference in clinical endpoints or serious adverse events could be observed [18].

## Bleeding risk or adverse effects in acute myocardial infarction

Inhibition of prostaglandin synthesis is responsible for both the beneficial anti-inflammatory and antiplatelet effects and adverse effects due to a loss of protective prostaglandin functions in the upper gastrointestinal tract and the kidneys. Gastrointestinal (GI) side effects of aspirin usually result from a loss of gastric mucosa protection by prostaglandin E2 and adverse effects are very common and dose dependent; however, even low-dose aspirin (50–75 mg) is not entirely free from side effects. The variety of GI symptoms include mild disorders such as nausea, heartburn, vomiting, and minor bleeding as well as epistaxis, peptic ulcers and severe GI bleeding [4].

In acute coronary syndrome patients, bleeding events are associated with worse outcome and an increased mortality risk [19]. Therefore, it is important to weigh the antithrombotic and anti-ischemic benefit against the bleeding risk and to establish additional strategies to reduce bleeding risk (pharmacologically for gastric protection and procedural measures such as radial access) in patients undergoing primary PCI for acute coronary syndrome [20, 21].

Longitudinal data over the last 24 years (from 1995 to 2018) from the SWEDEHEART registry [22] showed an increase in in-hospital bleeding (fatal, intracranial or bleeding requiring transfusions) in patients with acute myocardial infarction from 0.5% to 2% in the years 2005 and 2006 and a slight decrease afterwards to around 1.3% in the latest period due to more bleeding avoiding strategies during hospital stay (e.g., radial approach); however, the advent of more potent antiplatelet therapies resulted in an increase in out of hospital bleeding events. This led to a higher rate of recurrent hospital admissions for bleeding events from the GI tract, urogenital system, airways, eyes, ears or cerebral, which occurred in 2.5% in the early phase and in 4% in the late period of the study. Compared to patients with NSTEMI, patients with STEMI had a higher risk of bleeding events. In contrast to the increased bleeding events ischemic end points (e.g., myocardial infarction, cardiovascular death and stroke) decreased from 18.4% to 9.1%.

## Chest pain as the trigger for aspirin self-administration

### Chest pain as main symptom of acute myocardial infarction

The challenge of self-diagnosis of acute myocardial infarction—Do specific chest pain characteristics indicate acute myocardial infarction?

Non-traumatic chest pain accounts for 4.7% of visits to the emergency department in the USA [23] and only 5.1% of these suffer from an acute coronary syndrome.

In an observational study [24] chest pain as a reason to contact a general practitioner was present in 1.26%. Potentially life-threatening causes including myocardial infarction were diagnosed in 8.4% of these patients.

In the preclinical setting without additional diagnostic tools it is difficult to delineate cardiac from noncardiac chest pain and to confirm the diagnosis of acute myocardial infarction [25]. So how can we sufficiently rely on a patient’s description of acute chest pain? Is the symptom of acute chest pain sufficient to recommend a self-prescription of aspirin?

Non-recognizing symptoms of myocardial infarction leads to a delayed presentation to the hospital and to a delay of starting timely life-saving treatment. A cross-sectional study including a representative adult population in the USA [26] showed that 6% of the 25,271 individuals assessed using a questionnaire were unaware of even 1 single symptom out of 5 typical symptoms suggesting myocardial infarction (chest pain, shortness of breath, pain in arms or shoulders, lightheadedness or feeling weak and jaw, neck or back pain). Only half of the questioned adults were aware of all of the five possible symptoms of myocardial infarction. The awareness of symptoms differed between sociodemographic subgroups. Ongoing national education campaigns may improve rapid identification of signs and symptoms of myocardial infarction and could improve survival [27].

Whereas the awareness of typical symptoms of myocardial infarction in the general population seems to be acceptable, atypical symptoms may cause diagnostic uncertainties. In a systematic review of studies including more than 300,000 participants [28] that investigated general knowledge of symptoms of myocardial infarction, the awareness of typcial symptoms of myocardial infarction in the general population was very poor. This resulted in a treatment delay which was longer in women who report atypical symptoms more frequently compared to men.

A systematic review and meta-analysis of 120,988,548 individuals worldwide [29] showed ethnic differences in the knowledge of symptoms of myocardial infarction. Low-income countries reported a lower awareness of typical symptoms compared to high-income countries without any gender differences.

The current American College of Cardiology/American Heart Association (ACC/AHA) chest pain guidelines [23] describe symptoms with a higher probability of ischemic origin including central or retrosternal pain, pressure or tightness, exertional or stress related. In contrast, a sharp, fleeting, positional, pleuritic or shifting pain may indicate more probably a nonischemic underlying cause. Additionally, patient risk factors and age have to be taken into account.

### Balancing benefit and risk for early self-administration of aspirin for chest pain

Thus, why not (self-)prescribing aspirin in every patient with acute chest pain? Do we expect any harm because of misdiagnosis?

In some high bleeding risk conditions, such as aortic dissection, the intake of aspirin might increase the risk of bleeding and mortality [30, 31] during cardiac surgery. Data from the ASPREE trial in primary prevention [5] showed that aspirin is associated with a 60% increased risk of GI bleeding events in older patients; however, there was no increased risk of fatal bleeding events. These findings were confirmed in a meta-analysis [32]. A possible reason for no increase in fatal bleeding might be the unmasking of occult GI pathologies including cancer, by taking aspirin.

For many noncardiac chest pain conditions (e.g., chest wall pain, osteochondritis, pleuritis, pneumonia) without an increased bleeding risk after aspirin administration, early (self-)prescribing aspirin might have neutral effects without causing any harm. Furthermore, in the case of misdiagnosis discontinuation of aspirin intake probably prevents a meaningful increase of bleeding events. Thus, the potential harm of one single dose of aspirin often seems to be negligible, although evidence is scarce. Available evidence shows that prehospital aspirin is rarely associated with adverse outcome [33]. As new cyclooxygenase (COX) cannot be generated by platelets, the efficacy of aspirin lasts as long as the half-life of the platelets. Restoring normal platelet activity usually takes 10 days as 20% of platelets with normal COX activity are already sufficient to guarantee almost normal hemostasis [4]. Balancing the potential benefit of early administration of aspirin in patients with acute myocardial infarction, the potential harm with one single aspirin dose in the case of misdiagnosis seems to be low or negligible.

A recent US analysis [34] studied the benefit of self-administration of aspirin in acute chest pain by using a population simulation model. In a synthetic cohort of adults ≥40 years created from population data of the US Census 2019, the number of acute myocardial infarction cases with severe chest pain was estimated from a meta-analysis. The effect of self-administration of 325 mg aspirin within 4 h of severe chest pain (not only patients with acute myocardial infarction) was examined. The main outcome parameter was cardiovascular mortality. Safety was assessed by investigating mortality from GI or intracerebral bleeding events. Only patients with a definite diagnosis of acute myocardial infarction afterwards used uninterrupted aspirin. All other patients stopped aspirin treatment after the first single dose. This study estimated that out of more than 2 million adults ≥40 years in the USA in 2019 suffering from severe chest pain 351,493 had an acute myocardial infarction and 92,372 died. Early self-administration of aspirin could prevent 13,980 deaths from acute myocardial infarction; however, 963 deaths from aspirin-induced bleeding events have to be taken into account. This results in the prevention of 13,016 deaths with more benefit in older adults and in men. The average calculated cost of the aspirin tablet per years of life saved was 3.70 USD. Given the clear benefit of aspirin self-administration in acute chest pain over the risk of potential bleeding events, this “aspirin in the pocket” strategy may be considered in patients within 4 h onset of acute chest pain to reduce cardiovascular mortality.

## Glimpse into the future

Currently, another antiplatelet strategy for patients with acute myocardial infarction is under investigation. Antiplatelet therapies other than aspirin, including P2Y12 inhibitors result in either a delayed antiplatelet effect or have to be administered parenterally. Selatogrel, a novel reversible and selective P2Y12 inhibitor, has been developed [35] for immediate onset of action in the setting of acute chest pain. Selatogrel is administered subcutaneously and self-administration in patients with a recent history of myocardial infarction and symptoms suggestive of another acute myocardial infarction is currently being studied in the SOS-AMI randomized trial [36].

## Summary and conclusion

**Fig. 1:**
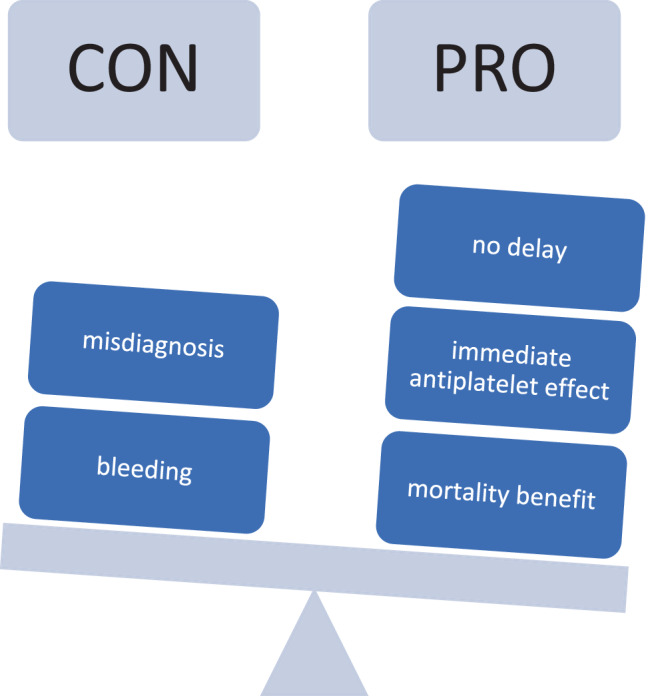
Balancing risks and benefits of prehospital aspirin administration

Aspirin is beneficial in patients with acute myocardial infarction resulting in reduced risk of cardiovascular mortality. “The earlier the better” strategy with aspirin use very early after the onset of acute chest pain should be considered for (self-)prescribing aspirin. Although there might be other underlying conditions causing chest pain in the absence of acute myocardial infarction, the benefit of reducing ischemic risk in the case of confirmed myocardial infarction outweighs the potential harm of one single dose of aspirin in patients without acute myocardial infarction (Fig. 1) Therefore, immediate self-administration of aspirin in patients suffering from acute chest pain represents a novel possibly life-saving therapeutic approach.
